# Whole genome analysis of *Yersinia ruckeri* isolated over 27 years in Australia and New Zealand reveals geographical endemism over multiple lineages and recent evolution under host selection

**DOI:** 10.1099/mgen.0.000095

**Published:** 2016-11-30

**Authors:** Andrew C. Barnes, Jerome Delamare-Deboutteville, Nicholas Gudkovs, Cara Brosnahan, Richard Morrison, Jeremy Carson

**Affiliations:** ^1^​ School of Biological Sciences, The University of Queensland, Gehrmann Laboratories (60), St Lucia, Brisbane, QL 4072, Australia; ^2^​ CSIRO Australian Animal Health Laboratory, Newcomb, VIC 3219, Australia; ^3^​ Ministry for Primary Industries, Animal Health Laboratory, Wallaceville, New Zealand; ^4^​ Department of Primary Industries Parks Water & Environment (DPIPWE), Kings Meadows, Launceston, TAS 7249, Australia

**Keywords:** *Yersinia ruckeri*, aquaculture, phylogeny, flagella, lipopolysaccharide, O-antigen

## Abstract

*Yersinia ruckeri* is a salmonid pathogen with widespread distribution in cool-temperate waters including Australia and New Zealand, two isolated environments with recently developed salmonid farming industries. Phylogenetic comparison of 58 isolates from Australia, New Zealand, USA, Chile, Finland and China based on non-recombinant core genome SNPs revealed multiple deep-branching lineages, with a most recent common ancestor estimated at 18 500 years BP (12 355–24 757 95% HPD) and evidence of Australasian endemism. Evolution within the Tasmanian Atlantic salmon serotype O1b lineage has been slow, with 63 SNPs describing the variance over 27 years. Isolates from the prevailing lineage are poorly/non-motile compared to a lineage pre-vaccination, introduced in 1997, which is highly motile but has not been isolated since from epizootics. A non-motile phenotype has arisen independently in Tasmania compared to Europe and USA through a frameshift in *fliI*, encoding the ATPase of the flagella cluster. We report for the first time lipopolysaccharide O-antigen serotype O2 isolates in Tasmania. This phenotype results from deletion of the O-antigen cluster and consequent loss of high-molecular-weight O-antigen. This phenomenon has occurred independently on three occasions on three continents (Australasia, North America and Asia) as O2 isolates from the USA, China and Tasmania share the O-antigen deletion but occupy distant lineages. Despite the European and North American origins of the Australasian salmonid stocks, the lineages of *Y. ruckeri* in Australia and New Zealand are distinct from those of the northern hemisphere, suggesting they are pre-existing ancient strains that have emerged and evolved with the introduction of susceptible hosts following European colonization.

## Data Summary

Sequence raw data (illumina reads, Pacbio movies), reference and draft genome assemblies and annotations are all deposited in GenBank and in the sequence read archive. These can be accessed collectively through bioproject PRJNA310959. (URL: http://www.ncbi.nlm.nih.gov/bioproject/?term=PRJNA310959).

Reference assembly accession numbers are also included in the manuscript text and in Table 1.

## Impact Statement

Aquaculture now supplies over half the world’s food fish and is a critical pillar in future food security. Beginning in the 1970s in Norway and Scotland, industrialized production of salmonids was initially beset by extensive losses to bacterial diseases resulting in high use of antibiotics but, with the advent of fish vaccination, the main diseases are now well controlled. Intensive salmon farming has dispersed to regions of the world that do not have native salmon populations. This combination of globalization and vaccine-induced immunity has applied selection to the population of aquatic pathogens. Salmonids were introduced to Australia and New Zealand around 150 years ago and substantial industries have recently developed in this biogeographically isolated region under conditions of strict inward biosecurity. Our study shows that *Yersinia ruckeri*, a major pathogen of farmed salmonid fish, has multiple genetically distinct lineages globally and within the Australasian region, but that the lineages in Australia and New Zealand are only distantly related to their northern hemisphere conspecifics, suggesting local endemism. Moreover, there has been independent evolution of a non-motile biotype 2 phenotype within Australia, and a lipopolysaccharide O-antigen-deficient serotype O2 phenotype has arisen independently on three occasions on three different continents.

## Introduction

*Yersinia ruckeri* is a member of the *Enterobacteriaceae* that causes enteric redmouth (ERM) disease in salmonid fishes and is endemic in cool temperate waters wherever salmonids are found (Barnes, 2011). Amongst the genus *Yersinia*, *Y. ruckeri* occupies a deep phylogenetic branch that supports *Y. entomphaga* and *Y. nurmii* and diverges from the lineage of the human pathogenic complex comprising *Y. pestis*, *Y. pseudotuberculosis* and *Y. similis* (Reuter *et al.*, 2014). In Australia, *Y. ruckeri* was first isolated in farmed rainbow trout (*Oncorhynchus mykiss*) in Victoria in 1963 and serological data indicated the likelihood of at least two serotypes at that time (Bullock *et al.*, 1978), although it is unclear whether these were recent (post-European colonization) introductions or pre-existed in the local environment. *Y. ruckeri* was isolated from farmed Atlantic salmon (*Salmo salar*) in Tasmania, Australia, in 1987 and has caused mortality in fish in hatcheries and post-seawater transfer in most years since. Vaccination against *Y. ruckeri* serotype O1b biotype 1 was introduced in Tasmania in the late 1990s with the development of Yersinivac-B based on an enzootic isolate of serotype O1b biotype 1. In 2007 the vaccine was modified by inclusion of a Tasmanian enzootic isolate of serotype O1b biotype 2. The bivalent vaccine has been broadly effective, particularly when administered parenterally. Isolation of *Y. ruckeri* from rainbow trout has been infrequent in Tasmania and the organism is not considered a major problem of trout species in Australia. In 2011 a putatively more virulent sorbitol-positive variant was isolated from hatchery-reared trout and in 2012 found in Atlantic salmon from the same hatchery; this variant has not since recurred. Historical serotyping, based on the scheme of Romalde *et al.* (1993), indicates that a single variant of the lipopolysaccharide (LPS O-antigen O1 serogroup, O1b, is present in Tasmania and New Zealand and distinct from the O1a Hagerman strain which is exotic to Australia and New Zealand. This probably reflects the geographical isolation, lack of migratory salmonids and strict inward biosecurity since the introduction of salmonids into Tasmania for aquaculture (Jungalwalla, 1991; Kahn *et al.*, 1999). In New Zealand, *Y. ruckeri* was first isolated from farmed Chinook salmon (*Oncorhynchus tshawytscha*) in 1989 and, while there are sporadic outbreaks in Chinook salmon, it is not considered a major problem and is often isolated from healthy fish (Anderson *et al.*, 1994). Consequently, vaccination against *Y. ruckeri* has not been required in New Zealand.

In Europe and the USA, infection with *Y. ruckeri* has predominantly been problematic in farmed trout and was consequently targeted by some of the earliest commercially available fish vaccines (Barnes, 2011; Busch, 1978). Vaccination employs formalin-killed bacterins delivered either by immersion in the hatchery or by intraperitoneal injection when the fish reach sufficient size (Tebbit *et al.*, 1981), which, at least until the turn of the century, had substantially reduced the impact of the disease in many countries. *Y. ruckeri* infections occur more frequently in Atlantic salmon hatcheries in Tasmania and latterly in recirculating Atlantic salmon hatcheries in Norway. Persistence after seawater transfer can occur, and therefore the longer duration of immunity afforded by intraperitoneal injection favours this route of delivery. Vaccination against *Y. ruckeri* is broadly O-antigen serotype specific (Tinsley *et al.*, 2011b), and there is a requirement for LPS O-antigen in killed vaccines for protection against ERM in trout (Ispir & Dorucu, 2014; Welch & LaPatra, 2016). Indeed, a recent study has suggested that the high antigenicity of O-antigen may account for the unusually high protection afforded by these vaccines when delivered by immersion (Welch & LaPatra, 2016). However, full protection and cross-protection is much more complex, with flagella antigens also implicated in early vaccination studies (Barnes, 2011). Indeed, the critical protective role of the flagella antigens is corroborated by re-emergence in previously vaccinated fish of non-motile biotype 2 strains of the same O-serotype (Deshmukh *et al.*, 2012; Fouz *et al.*, 2006; Tinsley *et al.*, 2011b). It is likely that selective pressure of host immunity against the highly antigenic flagellin proteins has driven parallel evolution of the non-motile biotype on several occasions globally in farmed salmonids (Tinsley *et al.*, 2011a; Welch *et al.*, 2011; Wheeler *et al.*, 2009). Indeed, biotype 2 variants of serotype O1b have occasionally been isolated from Atlantic salmon in Tasmania since the introduction of routine vaccination. This evolutionary route has also been followed by *Salmonella enterica* serovar Dublin where field isolates from vaccinated cattle lack motility and are aflagellate, but result in more invasive human infections (Yim *et al.*, 2014).

**Table 1.** *Y. ruckeri* isolates used in this study AU, Australia; NZ, New Zealand. FW, freshwater; SW, seawater. Biotype 1 reported motile, biotype 2 reported non-motile.

Isolate	Geographical origin	Year	Host	Notes	Accession number*
QMA0397	Tasmania, AU	1987	*Salmo salar*	Serotype O1b, SW	SRX1818442
QMA0398	Tasmania, AU	1988	*S. salar*	Serotype O1b, SW	SRX1818443
QMA0399	Tasmania, AU	1988	*S. salar*	Serotype O1b, FW	SRX1818454
QMA0400	Tasmania, AU	1988	*S. salar*	Serotype O1b, FW	SRX1818465
QMA0401	Tasmania, AU	1989	*Oncorhynchus mykiss*	FW	SRX1818476
QMA0402	Tasmania, AU	1989	*S. salar*	SW	SRX1818480
QMA0403	Tasmania, AU	1990	*S. salar*	Serotype O1b, FW	SRX1818481
QMA0404	Tasmania, AU	1990	*S. salar*	Serotype O1b, SW	SRX1818482
QMA0405	Tasmania, AU	1991	*S. salar*	Serotype O1b, FW	SRX1818483
QMA0406	Tasmania, AU	1991	*S. salar*	Serotype O1b, SW	SRX1818484
QMA0407	Tasmania, AU	1992	*S. salar*	Serotype O1b, SW	SRX1818444
QMA0408	Tasmania, AU	1993	*S. salar*	Serotype O1b, FW	SRX1818445
QMA0409	Tasmania, AU	1993	*S. salar*	Serotype O1b, SW	SRX1818446
QMA0410	Tasmania, AU	1995	*S. salar*	Serotype O1b, FW	SRX1818447
QMA0411	Tasmania, AU	1995	*S. salar*	Serotype O1b, SW	SRX1818448
QMA0412	Tasmania, AU	1996	*S. salar*	Serotype O1b, FW	SRX1818449
QMA0413	Tasmania, AU	1997	*S. salar*	Serotype O1b, FW, vaccine strain	SRX1818450
QMA0414	Tasmania, AU	1997	*S. salar*	Serotype O1b, SW	SRX1818451
QMA0415	Tasmania, AU	2000	*S. salar*	Serotype O1b, FW	SRX1818452
QMA0416	Tasmania, AU	2000	*O. mykiss*	FW	SRX1818453
QMA0417	Tasmania, AU	2000	*S. salar*	Serotype O1b, SW	SRX1818455
QMA0418	Tasmania, AU	2000	*S. salar*	Serotype O1b, FW	SRX1818456
QMA0419	Tasmania, AU	2001	*S. salar*	Serotype O1b, FW	SRX1818457
QMA0420	Tasmania, AU	2001	*S. salar*	Serotype O1b, FW	SRX1818458
QMA0421	Tasmania, AU	2002	*S. salar*	Serotype O1b, FW	SRX1818459
QMA0422	Tasmania, AU	2002	*S. salar*	Serotype O1b, SW	SRX1818460
QMA0423	Tasmania, AU	2002	*S. salar*	Serotype O1b, SW	SRX1818461
QMA0424	Tasmania, AU	2002	*O. mykiss*	FW	SRX1818462
QMA0425	Tasmania, AU	2003	*Salvelinus fontinalis*	Serotype O1b, FW	SRX1818463
QMA0426	Tasmania, AU	2004	*S. salar*	Serotype O1b, FW	SRX1818464
QMA0427	Tasmania, AU	2004	*S. salar*	Serotype O1b, biotype 2, SW	SRX1818466
QMA0428	Tasmania, AU	2004	*S. salar*	Serotype O1b, SW	SRX1818467
QMA0429	Tasmania, AU	2005	*S. salar*	Serotype O1b, FW	SRX1818468
QMA0430	Tasmania, AU	2005	*S. salar*	Serotype O1b, raffinose positive, FW	SRX1818469
QMA0431	Tasmania, AU	2007	*S. salar*	Serotype O1b, biotype 2, SW	SRX1818470
QMA0432	Tasmania, AU	2007	*S. salar*	Serotype O1b, biotype 2, FW	SRX1818471
QMA0433	Tasmania, AU	2007	*S. salar*	Serotype O1b, biotype 2, FW	SRX1818472
QMA0434	Tasmania, AU	2008	*S. salar*	Serotype O1b, FW	SRX1818473
QMA0435	Tasmania, AU	2009	*S. salar*	Serotype O1b, FW	SRX1818474
QMA0436	Tasmania, AU	2011	*O. mykiss*	Sorbitol-positive, FW	SRX1818475
QMA0437	Tasmania, AU	2011	*S. salar*	Sorbitol-positive, FW	SRX1818477
QMA0438	Tasmania, AU	2012	*S. salar*	Sorbitol-positive, FW	SRX1818478
QMA0439	Tasmania, AU	2014	*S. salar*	Serotype O1b, SW	SRX1818479
QMA0440	Tasmania, AU	2014	*S. salar*	Serotype O1b, FW	CP017236
AHL1	South Island, NZ	2002	*Oncorhynchus tshawytscha*	FW	SRX1818417
AHL2	NZ	1991	unknown	unknown	SRX1818418
AHL3	South Island, NZ	2004	*O.* *tshawytscha*	FW	SRX1818419
AHL4	South Island, NZ	2011	*O.* *tshawytscha*	Serotype O1b, FW	SRX1818420
AHL5	South Island, NZ	2014	*O.* *tshawytscha*	FW	SRX1818421
AHL6	South Island, NZ	2014	*O.* *tshawytscha*	FW	SRX1818422
AHL7	South Island, NZ	2014	*O.* *tshawytscha*	FW	SRX1818423
RS41	Wisconsin, USA	1979	*O. mykiss*	Serotype O1a, FW	GCA_001166725.1
CSF007-82	Idaho, USA	1982	*O. mykiss*	Serotype O1a, biotype 1, FW	GCA_000824965.1
ATCC 29473^T^	Idaho, USA	1961	*O. mykiss*	Serotype O1a, biotype 1, FW	GCA_000754815.1
Big Creek 74	Oregon, USA	1974	*O.* *tshawytscha*	Serotype O2, FW	GCA_001166725.1
OMBL4	Finland		unknown fish species	unknown	GCF_001172905.1
37551	Chile	2013	*S. salar*	Serotype O1b, SW	GCA_000737165.1
SC09	China	2008	*Ictalurus punctatus*	FW	GCA_000775355.1

*All sequence reads can be accessed directly under project SRP069734.

Protective efficacy of vaccines is dependent upon the correct antigens being incorporated in the vaccine. Therefore, it is essential for the purpose of mass vaccination to know what antigens are required, and to have a robust understanding of the diversity and stability of antigenic types in the hosts’ environment. Typing of *Y. ruckeri* is complex, with O-antigen serotyping being dominant, but completely dependent upon availability and consistency of accurate typing sera (Romalde *et al.*, 1993). Other methods of typing that have been attempted with varying degrees of success are outer membrane protein analysis (Davies, 1991) and more recently MLST (Bastardo *et al.*, 2012, 2015). Recent advances in sequencing technology, and increasing availability of bioinformatics tools with sufficient power to resolve large datasets, have advanced our understanding of the evolution and diversity of pathogenic bacteria, including fish pathogens (Brynildsrud *et al.*, 2014). Following the emergence of *Y. ruckeri* in 1987 and 1989 in Tasmania and New Zealand, respectively, curated collections of *Y. ruckeri* isolates have been maintained and provide a unique opportunity to investigate, by whole genome sequence analysis, the microevolution and diversity of *Y. ruckeri* in this biogeographically isolated region.

## Methods

### Bacterial strains and culturing.

Forty-four *Y. ruckeri* isolates, held freeze-dried or frozen at −80 °C and spanning 27 years since first isolation in Tasmania in 1987 until 2014, were chosen for sequencing from the culture collection at DPIPWE. The identity of the cultures had been established phenotypically using MicroSys E24 identification panels (Carson & Wilson, 2009). Additionally, seven isolates collected between 1991 and 2014 from New Zealand, were also included (Table 1). All isolates were originally collected from diagnostic samples from cases of disease in farmed fish or incidental isolates found during routine screening; the sample set did not therefore constitute an a priori designed epidemiological survey. Isolates for whole genome sequencing were stored at −80 °C in tryptone soya broth (TSB; Oxoid) containing 25 % glycerol and routinely cultured on tryptone soya agar (TSA; Oxoid) for 18–24 h at 25 °C. For liquid culture, isolates were grown in TSB with vigorous aeration at 25 °C. Sorbitol and lactose fermentation were determined on sorbitol MacConkey and MacConkey agar, respectively, as well as by MicroSys E24 phenotyping.

### DNA extraction and sequencing.

DNA was extracted from 5 ml mid-exponential cultures with the Qiagen DNeasy mini kit (Qiagen). DNA was quantified by Qubit fluorimetry (Invitrogen) and the quality and purity were checked by gel electrophoresis followed by amplification and sequencing of almost-complete 16S rRNA genes using universal primers 27F and 1492R (Amann *et al.*, 1995). Derived 16S rRNA gene sequences were assembled in Sequencher V5.2.2 and then analysed by blast n. Upon confirmation of identity and purity, 1 µg of DNA from each isolate (Table 1) was used for preparation of paired-end Nextera XT libraries for subsequent sequencing via an Illumina HiSeq2000 system (Australian Genome Research Facility, Melbourne). An aliquot from the most recent isolate in the collection (QMA0440) was dried by vacuum centrifugation (SpeedVac) for single molecule sequencing using Pacific Biosciences RSII platform with P4 chemistry (GATC Biotech). Following genome assembly, long-distance PCR followed by Sanger nucleotide sequencing was performed to confirm assemblies across phage tail fibre genes where Illumina read support for the PacBio assembly was low or absent. Long-distance PCR was carried out using LongAmp *Taq* polymerase [New England Biolabs (NEB)] following the manufacturer’s protocol employing the primers listed in Table S1 and a protocol of 30 cycles with denaturation at 94 °C for 15 s, annealing at 60 °C for 30 s and extension time of 10 min per cycle. Amplicons were analysed by gel electrophoresis, then cleaned using shrimp alkaline phosphatase and exonuclease 1 (NEB) prior to Sanger sequencing by the Australian Genome Research Facility.

### *De novo* assembly and annotation.

PacBio sequencing reads from two SMRT cells were assembled using the SMRT Portal version 2.3.0 (Pacific Biosciences) and the hierarchical genome assembly process (HGAP3), with read cut-off lengths set at 1500 bp. Assemblies were performed several times and polished using Quiver (Pacific Biosciences) (Forde *et al.*, 2014). Reads from the Illumina HiSeq2000 were initially trimmed using the clip function of the Nesoni package (http://www.vicbioinformatics.com/software.nesoni.shtml). As the Nextera XT libraries resulted in very high-quality sequence reads with no quality deterioration up to 125 bp when analysed using FastQC, but with some per-base sequence content ambiguity in the first 10–15 nt of each read, clip parameters were set to remove the first 15 bp with a minimum final read length of 75 bp. Assembly was performed using the SPAdes Assembler version 3.7.1 (Bankevich *et al.*, 2012), employing the - -careful and - -cov-cutoff 10 flags. Contigs were then re-ordered by alignment with the reference PacBio assembly using the move-contigs tool in the Mauve aligner (Rissman *et al.*, 2009). Genome annotation was performed using Prokka (Seemann, 2014) and then manually checked and curated with Artemis (www.sanger.ac.uk) (Carver *et al.*, 2012). Integrated prophages were detected using the PHAST server (Zhou *et al.*, 2011).

### Recombination and phylogenetic analyses.

To determine approximate phylogenetic relationships and provide a basis for analysis of recombination amongst the Tasmanian isolates and the few sequenced exotic isolates available through GenBank (Table 1), rapid alignment of core genomes was performed using parsnp in the Harvest Tools suite version 1.1.2 using the –c flag to include all genomes in the analysis (Treangen *et al.*, 2014). The resulting core genome alignment was exported for analysis for evidence of recombination using Gubbins (Croucher *et al.*, 2015). Approximate phylogenies for the global isolates were then inferred from post-filtered polymorphic sites, exported from Gubbins, by maximum-likelihood (ML) using RAxML 8.2.8 with the general time reversible nucleotide substitution model (GTR) and bootstrap support from 1000 iterations. To account for branch length distortion resulting from the choice of only variant sites, an ascertainment bias correction based on the number of known invariant sites across the genomes was applied using the -m ASC_GTRGAMMA - -asc-corr=felsenstein flags in RAxML (Leaché *et al.*, 2015; Stamatakis, 2014). To establish correct rooting of the trees, a replicate alignment including the draft genome of *Y. entomophaga* MH96 (GCA_001656035.1) as the out-group was conducted and phylogeny was inferred by ML using parsnp. Tree topologies were then compared.

To infer approximate divergence times amongst the Australian and New Zealand isolates where raw Illumina reads were available, high-quality SNPs were identified by read mapping. First, trimmed reads were mapped against the reference genome using the mapping tool in Geneious version 9.1 (Biomatters) with the medium sensitivity setting and five iterations. Reads were excluded that mapped to multiple regions, with an average PHRED score below 30, or with ambiguous base calls (Ns), as were unpaired reads (singletons). Next, variants were called using the Geneious variant caller with parameters set to exclude reads with paired-distance greater than 100 % above the expected insert size. The resulting SNPs and short indels were then filtered so that SNPs with quality less than 30, a frequency of less than 0.9, strand bias of greater than 65 % and coverage depth of less than 10 were excluded. After manual curation around large insertions in Geneious, SNPs and small indels were used to create ‘pseudogenomes’ by writing into the reference genome. The resulting 50 pseudogenomes plus the reference genome were then exported as an alignment to Gubbins for inference and removal of regions of recombination. The output filtered core genome polymorphic sites were then used to infer genetic distance between isolates by ML using RAxML with parameters and ascertainment bias correction applied as above.

The presence of a temporal signal was investigated in trees derived from all exotic isolates and separately amongst the Tasmanian O1b lineage by performing a root-to-tip regression analysis of both the unrooted and the best-fitting rooted tree from RAxML using TempEst (Rambaut *et al.*, 2016), and an approximate estimate of substitution rate over time derived from the slopes for each case. Then, the temporal relationship amongst exotic strains and separately within the Tasmanian O1b lineage was analysed by Bayesian inference using Beast 2.3.2 (Bouckaert *et al.*, 2014). To optimize model parameters, several substitution models and population prior assumptions were tested in Beast. The gamma site model was used with gamma category count set to four and the proportion of invariant sites fixed at the number of SNP positions divided by the genome length. The Hasegawa–Kishino–Yawa (HKY) and GTR substitution models were tested with relaxed log normal, strict and random local clock models. The coalescent exponential, constant and Bayesian skyline population priors were employed with the uncorrelated log normal clock distribution (UCLD) set to gamma where required, and the Markov Chain Monte Carlo method was run for 100 million generations, sub-sampling every 5000 iterations. Traces were compared in Tracer 1.6.0. The HKY substitution rate model with four gamma categories, a strict clock and coalescent exponential population model was found to give the lowest Akaike information criteria (AIC) scores in Tracer. Therefore, this model was run three times with differing random seed values and minor tuning of the scale operators between runs for 100 million generations of the Markov chain, sampling every 5000 steps. A maximum clade credibility tree based on the median tree height was generated using Tree Annotator, which was viewed and annotated in FigTree V1.4.2. To ascertain that resulting trees were driven largely by the data rather than the prior assumptions, a replicate model was run sampling only from the priors.

### Pan-genome assembly.

To facilitate genome comparison, including non-core regions, a pangenome was assembled using GView (Petkau *et al.*, 2010) from three complete closed reference quality genomes [QMA0440, this study; CSF007-82 (Nelson *et al.*, 2015); and Big Creek 74], and curated draft genomes were then added sequentially. The resulting pangenome was used as a reference for complete multiple genome blast comparison and viewing using BRIG (Alikhan *et al.*, 2011). To investigate conserved core genes, coding DNA sequences from all strains were analysed with Roary (Page *et al.*, 2015), using default settings.

### Macroscopic agglutination assay.

Bacterial isolates were grown in parallel on Oxoid blood agar base no. 2 with 7 % defibrinated sheep’s blood at 21 °C in air for 3 days prior to testing. The macroscopic agglutination reaction was assessed on polished glass microscope slides at ambient temperature (23 °C). Four bacterial isolates were tested on each slide incorporating reference and query isolates such that four separate spots of 20 µl of diluted antiserum (Table S3) were applied along the slide and a small amount of growth was emulsified using a 1 µl loop. Slides were gently rocked to facilitate mixing and examined by eye for signs of agglutination. Slides were examined for up to 3 min or until evaporation affected the flow of liquid.

### Motility assays.

Swimming and swarming motility were determined in 90 mm Petri dishes using swim agar and swarm agar, respectively, as previously described (Atkinson *et al.*, 2006), with some modifications. Briefly, both agars comprised a base containing 10 g peptone (Oxoid), 3 g yeast extract (Oxoid) and 5 g NaCl per litre. Agar was included at 6 g l^–1^ in swarm agar and 3 g l^–1^ in swim agar. Additionally, for swarm agar, 15 g glucose was added aseptically from a 50 % (w/v) sterile solution prior to pouring the plates. Swim agar was inoculated by touching a single colony (O/N TSA, 25 °C) with a sterile inoculating needle and stabbing into the medium. Swarm agar was similarly inoculated except that the needle was touched on the surface of the medium. Plates were incubated for 48 h at 25 °C. Swimming and swarming motility were scored based on the diameter of the growth.

### LPS preparation and analysis.

LPS was extracted from cells from 10 ml of overnight lysogeny broth (LB; Oxoid) culture (25 °C) using the tri-reagent method of Yi & Hackett (2000). LPS was separated by electrophoresis in a 12 % acrylamide SDS-PAGE gel at 180 V for 90 min. Gels were fixed in 30 % ethanol/10 % acetic acid for 5 min then oxidized with 0.7 % sodium metaperiodate in fixative for 15 min based on the method of Tsai & Frasch (1982). The gels were then stained with silver using a commercial kit (Pierce Silver Staining Kit; Thermo Fisher), photographed and dried.

### Transmission electron microscopy (TEM).

A single colony was picked from an overnight LB agar culture and suspended in a drop of ultrahigh-purity water. Glow-discharged carbon and Formvar-coated copper grids were placed on drops of bacterial suspension for 2 min. Most of the solution was blotted away before the grids were negatively stained with 1 % uranyl acetate and then blotted dry fully. The grids were examined under a JEOL 1010 transmission electron microscope operated at 80 kV. Micrographs were captured using an analySIS Megaview III digital camera.

### Flagella operon RT-PCR.

LB cultures (10 ml) were grown to mid-exponential phase (approx. 5 h at 25 °C) from a 2 % (v/v) inoculum taken from an overnight LB culture. Cells were harvested (4000 ***g***, 10 min, 4 °C), resuspended in 1 ml cold nuclease-free water and divided equally between two microcentrifuge tubes. One tube was used for DNA extraction (DNAeasy; Qiagen) whilst 1 ml RNAprotect (Qiagen) was added to the second tube and processed for RNA extraction (RNeasy; Qiagen), with an on-column DNAse treatment to remove any residual genomic DNA (RNase-free DNase set; Qiagen). DNA and RNA were quantified by Qubit fluorometry (Invitrogen) and 2 ng RNA was reverse transcribed using the VILO Reverse Transcription kit (Invitrogen). For PCR and RT-PCR, 40 ng gDNA and 2 µl cDNA, respectively, was used in each reaction. PCR primers (Table S1) were designed such that all reactions ran under the same protocol, comprising 1 min at 95 °C, then 30 cycles at 95 °C for 15 s, 58 °C for 30 s, 72 °C for 30 s followed by a final extension at 72 °C for 7 min. Amplicons were analysed by electrophoresis at 80 mA in 2 % agarose gels containing 0.01 % Hydragreen dye (ACTGene).

## Results

### Complete genome assembly of *Y. ruckeri* QMA0440 serotype O1b

Assembly of the reference quality *Y. ruckeri* QMA0440 genome using HGAP3 from 126 882 post-filtered reads derived from two SMRT cells yielded a single contig of 3 866 096 bp with 117-fold coverage. Analysis for both internal and external self-complementarity using Contiguity (version 1.04) (Sullivan *et al.*, 2016) indicated no internal self-complementary regions, but complementary overlapping regions were detected at each end. Consequently, 9462 bp was manually trimmed and the genome was closed with a final chromosome size of 3 856 634 bp and G+C content of 47.7 mol%. To check the accuracy of the closed chromosome, trimmed paired-end reads from a Nextera XT library prepared from the same DNA sample and sequenced via the Illumina HiSeq2000 system were aligned using SHRiMP2 and uniformity of coverage was checked by visualizing in Tablet (Milne *et al.*, 2010, 2013) and Artemis. A total of 4 474 297 reads were mapped and a consensus sequence was generated to assess any differences between the PacBio and Illumina data. The resulting alignment revealed a single 2 nt deletion and a single 2 nt insertion, both in tandem repeat regions, in the Illumina sequence. However, Illumina read coverage across a putative integrated prophage in the PacBio assembly was low or absent. Therefore, this section of the assembly was checked by amplifying across the 45 kb region in fragments of 7–12 kb by long-distance PCR followed by Sanger sequencing. This confirmed the accuracy of the PacBio assembly and the genome was considered closed. The ends were joined and the resulting sequence was re-cut immediately upstream of the *dnaA* chromosomal origin of replication. This linear chromosome was then annotated with Prokka using the *Yersinia* species database. Further manual curation was undertaken with Artemis.

### Draft assembly of 43 Tasmanian and seven New Zealand *Y. ruckeri* isolates

The Illumina paired-end reads from the remaining 43 Tasmanian isolates and seven New Zealand isolates were assembled with SPAdes version 3.7.1 resulting in contig numbers ranging between 50 and 135 and genome sizes ranging between 3 647 605 and 3 897 373 bp (Table S2). Seventeen isolates sequenced in the first sequencing batch comprising isolates QMA0397 to QMA0414, except QMA0413, contained some contamination from the internal standard phi X174. However, these reads assembled into a single contig of 5441 bp in each case, allowing post-assembly removal. Mean coverage (by mapping) ranged between 377- and 513-fold in the first 17 isolates sequenced in a single illumine flow cell, and between 37- and 137-fold in the remainder that were sequenced as a 96-plex batch.

### Phylogenetic analysis

**Fig. 1.**
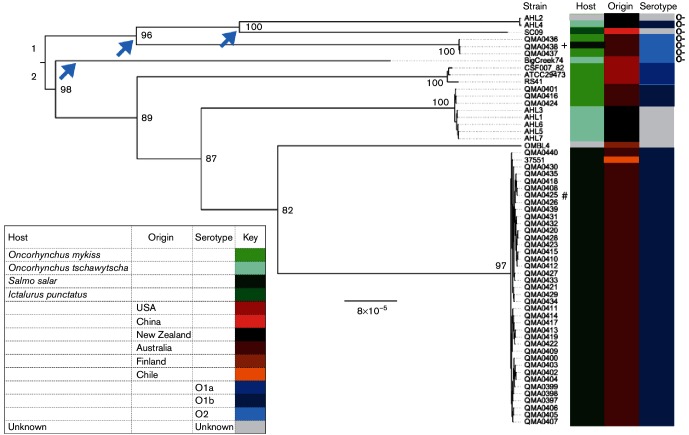
ML phylogenetic tree of 58 *Y. ruckeri* isolates. The tree was based on alignment of 11 715 non-recombinant core genome SNPs with branch lengths corrected for ascertainment bias using RAxML. Branch support was estimated by performing 1000 bootstrap replicates. Metadata show host, geographical origin and serotype where known. Bar, estimated substitution rate. + Isolate collected from Atlantic salmon (*S. salar*) reared concurrently with rainbow trout (*O. mykiss*) in the same hatchery. # Isolated from brook trout (*Salvelinus fontinalis*) reared alongside Atlantic salmon in the same hatchery. Arrows indicate independent losses of the LPS O-antigen cluster and O-antigen-deficient isolates are indicated by O-.

A total of 11 715 non-recombinant core genome SNPs and small indel positions were identified from whole genome sequence alignments of the 44 Tasmanian isolates and seven New Zealand isolates with genome sequences of six isolates from other countries obtained from GenBank (Table 1, Fig. S2). ML analysis using RAxML indicated two deep branching genetic lineages (Fig. 1). Lineage 1 comprised isolates from Tasmania, New Zealand and China, with deep branches segregating the isolates from different countries (Fig. 1). Lineage 2 comprised isolates from the USA, Tasmania, New Zealand and a single isolate each from Chile and Finland (Fig. 1). Within lineage 2, phylogenetic clustering occurred predominantly along geographical lines with two notable exceptions. First, serotype O1b isolates from Atlantic salmon, *S. salar*, in Australia clustered with one from Chile, also isolated from *S. salar*. Secondly, five isolates from Chinook salmon in New Zealand (AHL1, 3, 5, 6, 7) clustered with rainbow trout isolates from Australia (QMA0401, 416 and 424) that have been associated with only minor losses, suggestive of low virulence in respect of the host species. This cluster composition suggests a recent common origin for these Tasmanian and New Zealand isolates (Fig. 1). Clustering amongst serotype was more cryptic as not all strains had been typed. However, the serotype O1a strains grouped separately from the serotype O1b strains. However, the typed serotype O2 strains, which lack the O-antigen biosynthesis operon, occupied multiple branches. Indeed, the loss of O-antigen cluster has occurred on three separate occasions amongst the isolates analysed (Fig. 1, arrows).

**Fig. 2.**
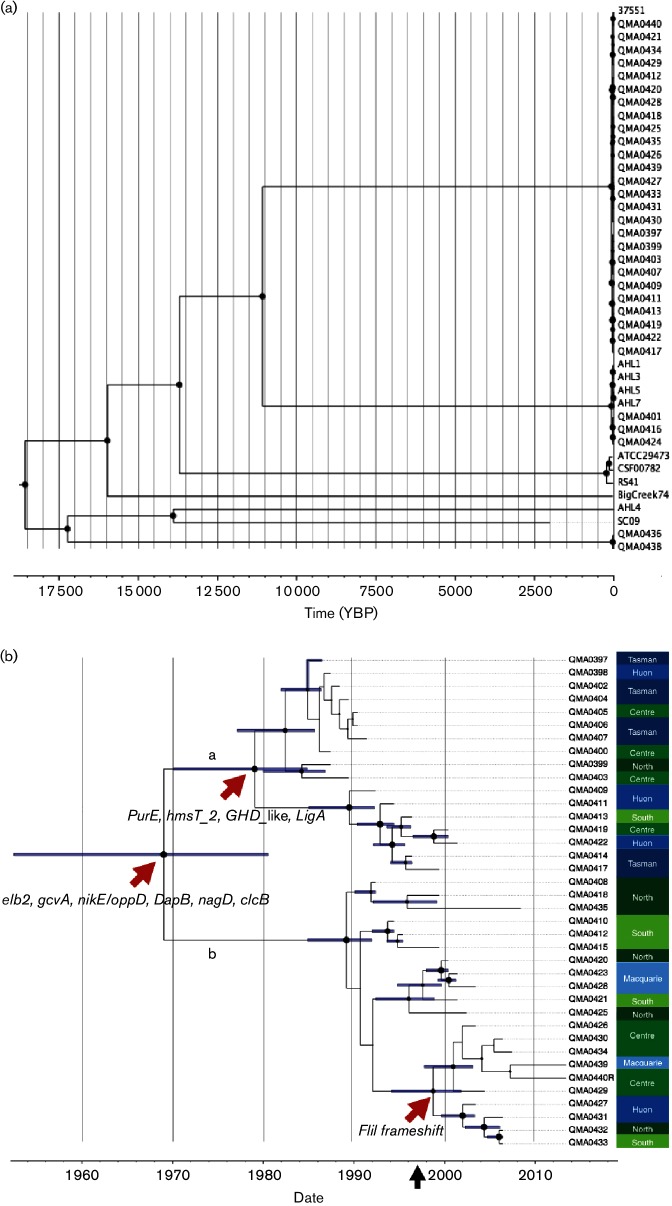
Time-calibrated evolution of *Y. ruckeri* lineages. (a) Evolution of global lineages of *Y. ruckeri* based on alignment (parSNP) of 11 088 non-recombinant core-genome SNPs. Isolates without accurate sample date, or where there was polytomy amongst the Tasmanian O1b isolates, were removed prior to analysis resulting in alignment of 42 isolates spanning 53 years. The *x*-axis indicates estimated divergence date in number of years before present. (b) Evolution of the Atlantic salmon O1b lineage in Tasmania based on alignment of 63 non-recombinant SNPs from 38 isolates spanning 27 years. Red arrows indicate non-synonymous SNPs at subclade nodes. The black arrow (*x*-axis) indicates introduction of vaccination against *Y. ruckeri* O1b in Atlantic salmon in Tasmania. The *x*-axis represents estimated date whilst the horizontal bars represent 95 % highest posterior density (HPD). For both phylograms, a time-calibrated phylogeny was estimated by Bayesian inference with beast 2.1.3 using the HKY substitution model with four gamma categories, a strict clock and coalescent exponential population priors. Trees represent maximum clade credibility based on the median height derived from 100 million iterations with trees sampled every 5000 iterations. In both cases, node circle size is proportional to posterior probability of the branch being supported (minimum 0.5). Colour-coded regions of production are on the right *y*-axis; blue labels are marine sites and green are freshwater hatcheries.

Dating the divergence of the various lineages of the collection of isolates using Bayesian inference suggested a most recent common ancestor (MRCA) originating around 18 500 years before present (YBP), but over that timescale the 95 % highest posterior density (HPD) was rather broad (12 355–24 757 YBP). The O1b lineages that are predominant in Australia and New Zealand share an ancestor with the O1a lineages from the USA and diverged around 13 500 YBP (HPD, 9048–20 545) (Fig. 2a). Within the O1b lineages, the virulent Atlantic salmon isolates diverged from the lower virulence rainbow trout (Australia) and Chinook salmon isolates (New Zealand) 10 890 YBP (7265–16 756) (Fig. 2a), whilst amongst these lower virulence isolates the Australian and New Zealand strains have an MRCA dated around 68 years ago (43.9−106.1). Interestingly, strain 37 551, O1b isolated in Chile in 2011, shared an MRCA with the reference isolate QMA0440 and diverged between 4 and 34 years ago (Fig. 2a).

Only 63 non-recombinant SNP and short indel positions across the core genome described the diversity amongst the Tasmanian serotype O1b Atlantic salmon isolates over the 27-year time span of the collection. To gain greater resolution amongst this lineage, further Bayesian analysis was conducted and revealed two distinct clades with a common ancestor around 1969 (1952–1981) (Fig. 2b). Clade (a) comprises highly motile phenotypes, but there is a subclade of low-motility isolates arising in the late 1970s and coinciding with the establishment of the Atlantic salmon industry in Tasmania. Clade (a) appears to have become extinct with no isolates derived from this lineage since 2002. In contrast, clade (b) contains most of the recent isolates, including the non-motile biotype 2 phenotype, which share an MRCA dated around 1997 with the reference isolate (QMA0440) and coincident with the introduction of mass vaccination against *Y. ruckeri* in Tasmanian Atlantic salmon with a vaccine based on serotype O1b biotype 1 (Fig. 2b). Whilst there was some evidence of localized endemism, for example the subclade containing QMA0408, 418 and 435 came from a single hatchery with strains isolated between 1993 and 2009 (Fig. 2b, Table 1), this was not generally evident across farm sites and reflects the nature of salmonid farming in which animals are moved between freshwater hatcheries and different marine cage sites during their lifecycle (Fig. 2b).

### The *Y. ruckeri* pangenome

**Fig. 3.**
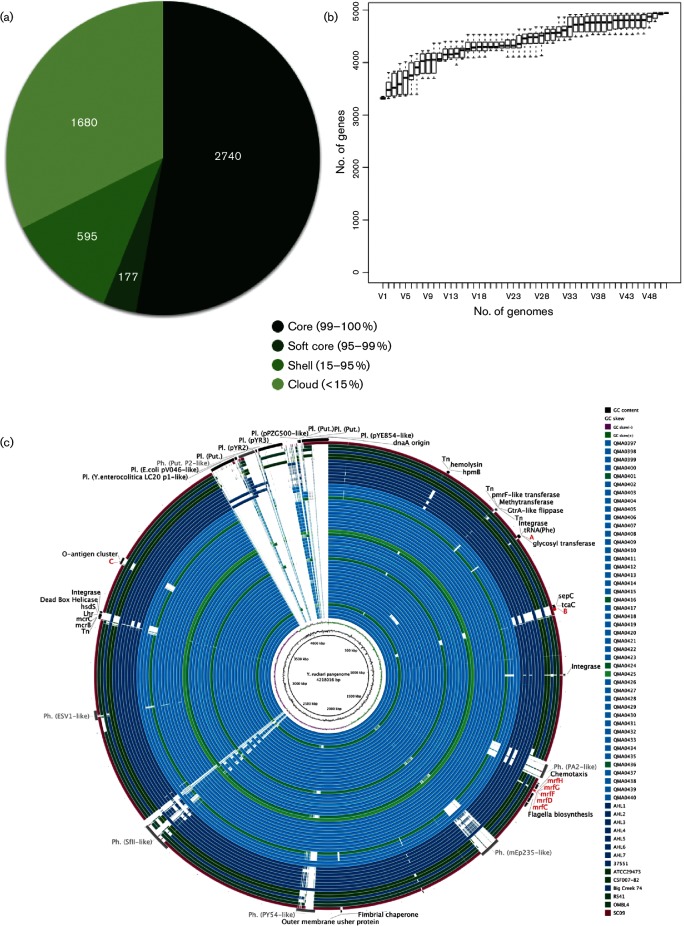
The *Y. ruckeri* pangenome. (a) Proportion of gene coding sequences (CDS) in the core, soft core, shell and cloud genome of *Y. ruckeri* based on analysis of assemblies of 58 isolates using Roary. (b) Unique gene accumulation with additional genomes. (c) A blast n-based sequence comparison of 58 *Y. ruckeri* strains against the *Y. ruckeri* pan-genome reference generated using GView server. The image was generated using BRIG (Alikhan *et al.*, 2011), with an e-value cut-off set at 10^–15^. Rings from the innermost to the outermost represent G+C content and G+C skew of the pan-genome reference, then sequence similarity of each of the 58 isolates listed in the legend, from top to bottom. Rings are coloured according to host, with Atlantic salmon strains in light blue, Chinook salmon isolates in dark blue, rainbow trout isolates in dark green, the single brook trout isolate in light green and the catfish isolate in purple. The last ring shows regions of differentiation between isolates such as plasmids, prophage and differentially present gene clusters (see text).

A coding sequence (gene)-based pangenome using Roary comprised 4943 genes with a core genome of 2745 genes (Fig. 3a), but is not considered closed as new genes were continuing to be added with the addition of each new sequenced genome (Fig. 3b). A complete nucleotide sequence-based pangenome created from 58 genomes with GVIEW server and then analysed using BRIG revealed very high conservation amongst the lineages, with a total length of 4 218 016 bp compared to the reference genome of 3 866 096 bp (Fig. 3c). Indeed, the majority of the difference between the genomes was explained by mobile genetic elements. Plasmids pYR2 and pYR3 were exclusive to strains RS41 and ATCC 29473. Type IV secretory components were identified by Hidden Markov Model (HMM) during annotation, probably also plasmid-mediated, in CSF007-82, RS41 and SC09. In SC09, these were assembled into a single contig in the draft genome along with a cluster of conjugal transfer proteins suggestive of a plasmid, although it was not possible to determine which without availability of supporting long read data (Fig. 3c).

**Fig. 4.**
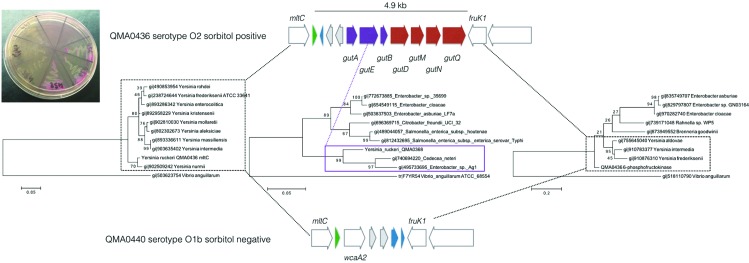
Horizontally acquired sorbitol utilization cluster from O2 serotype *Y. ruckeri* isolates. The inset photograph shows sorbitol utilization on sorbitol MacConkey agar by three newly identified O2 isolates (clockwise from the right: QMA0436, 437 and 438) from Tasmania, compared to representative O1b isolates (clockwise from the bottom: QMA0435, 440 and 439). ML phylogenetic trees were constructed with mega version 6.06 based on a muscle alignment of amino acid sequences from closest matching species recovered by blastp from NCBI. Bootstrap support for branches was derived from 200 replicates. The sorbitol-specific PTSII transporter subunits are indicated in purple. Co-acquired genes are indicated in red with tRNA in green and putative transposes in grey.

Major differences were found in the acquisition of phages throughout the genome, with the Tasmanian serotype O1b Atlantic salmon isolates generally containing more integrated prophages than the Tasmanian trout isolates or those from outside Australasia (Fig. 3c). For example, a PY54-like intact prophage was present exclusively in all Tasmanian Atlantic salmon serotype O1b isolates, but absent from the exotic isolates and the Tasmanian sorbitol-positive types. Similarly, remnants of a putative prophage also accounted for the sorbitol-positive phenotype of the recently isolated Tasmanian O2 serotypes. Occurring as a complete phosphotransferase system (PTS) sorbitol-specific transport complex, it is located between phage integrases immediately adjacent to a tRNA and lying between the murein transglycosylase *mltC* and a 6-phosphofructokinase *fruK1* that were conserved through all isolates in this study (Fig. 4). The sorbitol PTS membrane-associated transport complex comprised the *gutA*, *gutE* and *gutB* components, which by blast, and corroborated by phylogenetic analysis, show closest similarity to proteins found in *Cedecea neteri* and *Enterobacter* sp., other species of the *Enterobacteriaceae* not in the genus *Yersinia* (Fig. 4). In contrast, the conserved flanking genes, *mltC* and *fruK1*, shared highest similarity with other members of the genus *Yersinia*, indicating that the sorbitol-specific system was probably acquired by horizontal gene transfer. This configuration was conserved in the serotype O2 Big Creek 74 isolate from Oregon and SC09 from China in spite of the distant phylogenetic relationship between these isolates (Figs 1 and 4); this arrangement, however, was absent from the New Zealand AHL2 and AHL4 isolates from the same lineage. In the sorbitol-negative isolates from Tasmania, the predominant phenotype, the *gut* complex was absent but a glycosyl transferase similar to *wca* from streptococci and two additional transposases were located between the *mltC* and *fruK1* genes (Fig. 4). Interestingly, this was the same configuration found in this region of AHL2 and AHL4 from New Zealand, although these isolates were more closely related to the O2 lineage (Fig. 1).

**Fig. 5.**
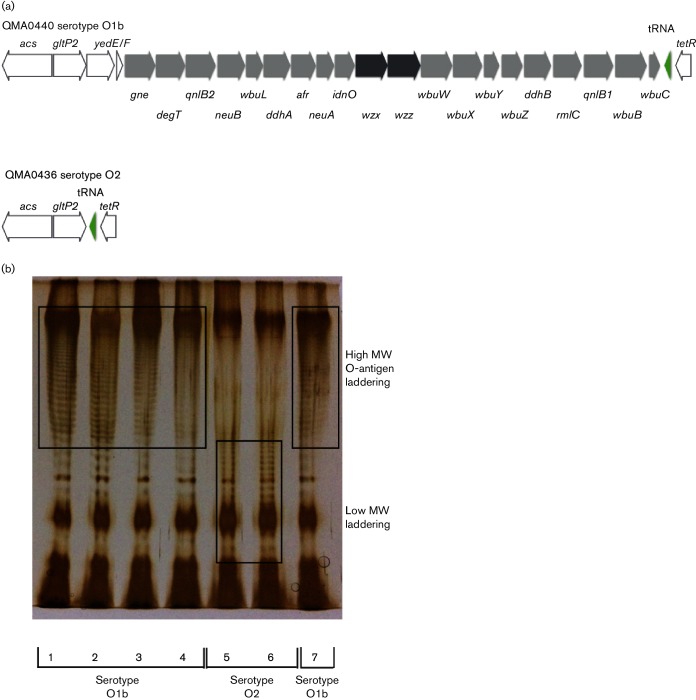
LPS O-antigen biosynthesis in O1b and O2 strains of *Y. ruckeri*. (a) Gene order (trailing strand) in reference genome of QMA0440 (O1b) and QMA0436 (O2). Pale grey shows genes encoding polysaccharide-modifying enzymes derived from the QMA0440 genome annotation. The flippase and polymerase are shown in dark grey and adjacent tRNA in green. (b) SDS-PAGE gel (12 % acrylamide) showing purified LPS from O1b isolates QMA0401 (1), QMA0404 (2), QMA0424 (3), QMA0432 (4) and QMA0440 (7) and O2 isolates QMA0436 (5) and QMA0438 (6). Gels were stained with silver after initial oxidation with sodium periodate according to the method of Tsai & Frasch (1982).

Within the chromosome, deletion of the O-antigen cluster in the serotype O2 Big Creek 74 strain was also evident in SC09 from China and three recently isolated sorbitol-positive isolates from a single hatchery producing salmon and rainbow trout in northern Tasmania in 2011 and 2012 (Table 1, Figs 3c and 5a). The O2 serotype for these three isolates was confirmed retrospectively by a macroscopic agglutination assay (Table S3). The flippase-dependent O-antigen cluster comprised 20 genes mapping between a *tetR*-like transcriptional regulator and *gltP*-like proton symporter (Fig. 5a, Table S4) and was conserved amongst the O1b and O1a isolates sequenced (Fig. 3c). Analysis of purified LPS from Tasmanian isolates by SDS-PAGE clearly showed the absence of high-molecular-weight O-antigen laddering in the sorbitol-positive O2 serotype strains (Fig. 5b). Although the New Zealand isolates AHL2 and AHL4 clustered with these serotype O2 isolates, albeit with very deep branches, both had complete O-antigen biosynthesis clusters within the genome (Figs 1 and 3c). In OMBL4 from Finland, general secretory pathway proteins M to K were deleted in a large cassette that also included all of the type III secretion system proteins (Fig. 3c). Strain QMA0406 of the O1b Tasmanian Atlantic salmon lineage lacked siderophore biosynthesis and uptake proteins for yersinibactin and enterobactin siderophores in a 70 kb deletion that also included all the genes of the tight adherence operon (Fig. 3c). The low-virulence isolates from rainbow trout in Australia, QMA0401, 416 and 424, lacked the large mobile genetic element containing putative insecticidal toxins *sepC* and *tcaC* associated with a transposon that was also lacking in New Zealand isolates AHL1, 3, 5, 6 and 7 and the USA O1a serotypes and Finnish isolate OMBL4 (Fig. 3c). These Tasmanian rainbow trout isolates, along with the New Zealand isolates in the same clade, were also missing a major haemolysin (Figs 1 and 3a). The serotype O1a strains from the USA lacked the *mrfDFGH* pilin export gene cluster that was present in all other sequenced serotypes (Fig. 3c).

**Fig. 6.**
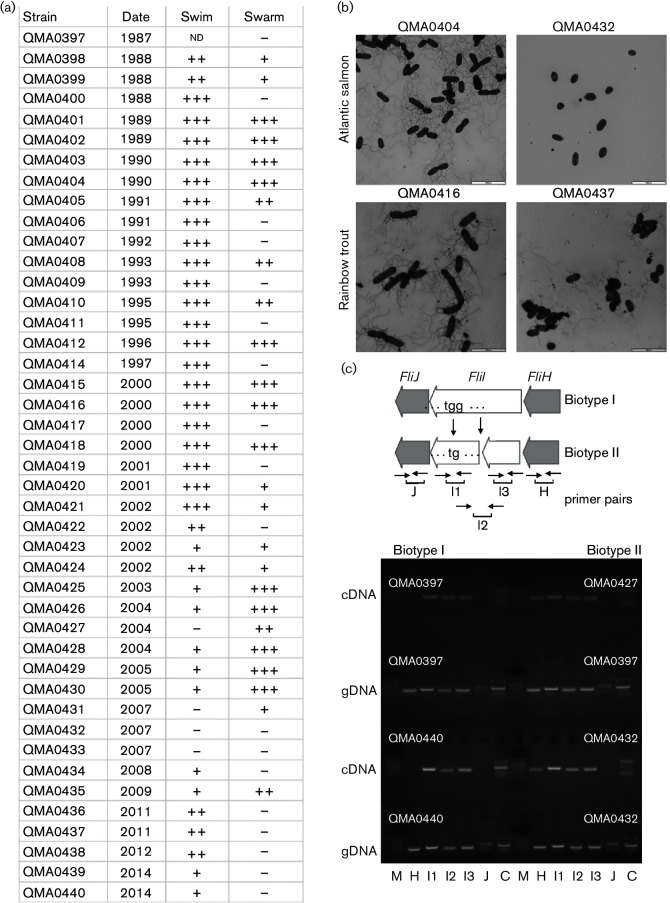
Motility in Tasmanian *Y. ruckeri* isolates and genetic basis for biotype 2 in Tasmania. (a) Swimming and swarming motility determined on swim or swarm agar after 24 h at 25 °C. (b) TEM of isolates QMA0404 (O1b salmon, biotype 1), QMA0432 (O1b salmon biotype 2), QMA0416 (O1b rainbow trout, motile) and QMA0437 (O2 rainbow trout, motile) negatively stained with 1 % uranyl acetate. Cells were examined on Formvar-coated grids with a JEOL 1010 microscope at 80 kV, and captured with an analySIS Megaview III digital camera. Bars, 5000 nm. (c) Effect of frameshift mutation in *fliI* and on transcription of surrounding genes in the *Y. ruckeri* flagella gene cluster. The positions of primers are indicated. Agarose (2 %) gel electrophoresis of genomic DNA and cDNA from two biotype 1 (QMA0397 and 440) and biotype 2 (QMA0427 and 432) isolates from Tasmania. Lanes: M, marker; H FliH_F/FliH_R; J, FliJ_F/FliJ_R; I1, FliI_indF/FliI_indR amplified across indel in *fliI*; I2, FliI_shortF/FliI_shortR amplified truncated fragment upstream of indel; I3 FliI_longF/FliI_longR amplified fragment downstream of indel; C, two multiplexed FliC primer pairs (refer to Table S1 for primer information).

Swimming motility of *Y. ruckeri* isolates from Tasmania declined from 2002 onwards (Fig. 6a). Four isolates were reported to be completely non-motile biotype 2 by DPIPWE during first isolation (Table 1). These isolates produced no flagella when assessed by TEM (Fig. 6b) and shared a frameshift mutation in *fliI* resulting in an early stop codon in the gene product, a component of the rotating ATPase complex required for translocation of the flagellar assembly through the membranes (Fig. 6c). To determine whether the frameshift resulted in polar effects in the operon, RT-PCR was used to investigate transcription of *fliH* and *fliJ* in addition to *fliI* using primers upstream and downstream of the frameshift, as well as across the frameshift mutation (Fig. 6c). Two biotype 2 strains were compared with two biotype 1 strains with genomic DNA as a positive control for primers and non-reverse transcribed RNA as a negative control. The RNA was transcribed across the frameshift with both upstream and downstream primer sets resulting in amplicons of the correct size in RT-PCR (Fig. 6c). Interestingly, *fliJ* was not transcribed in either biotype 1 or biotype 2 isolates, suggesting that under these culture conditions it is not transcribed as part of an operon. In contrast, *fliH* was not transcribed in biotype 1 isolates, but was transcribed in biotype 2 with the frameshift mutation, suggesting the interruption of a transcriptional termination signal by the deletion (Fig. 6c).

## Discussion

The genus *Yersinia* is a diverse group of species comprising pathogenic and non-pathogenic environmental enterobacteria of substantial animal and human health interest. *Y. ruckeri* is a pathogen of predominantly salmonid fish and, along with *Y. entomophaga* and *Y. nurmii*, clusters on very deep branches of a phylogenetic lineage that comprises the pathogenic *Y. pestis*, *Y. pseudotuberculosis* and *Y. similis* (Reuter *et al.*, 2014). The present study represents the first large-scale whole genome analysis of *Y. ruckeri* and reveals that, even within the species, there are several deep branching lineages with a common ancestor dated around 18 500 YBP. There is good evidence of geographical endemism amongst the *Y. ruckeri* isolates examined in this study. Only isolate 37551 from Chile was similar to the Atlantic salmon O1b isolates from Australia. The first report in Chile of yersiniosis in Atlantic salmon was made in 1992 (Toledo *et al.*, 1993). The disease was managed successfully through the use of ERM vaccines until 2008 when vaccinated Atlantic salmon developed yersiniosis (Bastardo *et al*., 2011). The serotype of isolates from these epizootics was found to be O1b (Bastardo *et al.*, 2011), the same serotype as that of isolate 37551 (Navas *et al.*, 2014). Between 2005 and 2012, iodine-disinfected eyed-eggs of Atlantic salmon were imported by Chile from Tasmania, a period of time coincidental with the reported appearance of serotype O1b in Chile. *Y. ruckeri* has been isolated from disinfected unfertilized Chinook salmon eggs (Sauter, 1986), and DNA of *Y. ruckeri* has been detected in unfertilized eggs and ovarian fluid of Chinook salmon (Glenn *et al.*, 2015). Evidence for eggs as a vector of the viable organism has yet to be shown conclusively, but the emergence of ERM in Venezuela is believed to have occurred through the importation of infected eggs (Furones *et al.*, 1993), which suggests they could be an unwitting means of *Y. ruckeri* transmission.

Tasmanian rainbow trout (*O. mykiss*) isolates form a genetic lineage that also includes isolates from Chinook salmon (*O. tshawytscha*) in New Zealand that is distinct from the Atlantic salmon O1b isolates, with an MRCA estimated between 49 and 106 years ago. It is therefore possible that this lineage was transferred with rainbow trout eggs from New Zealand in 1898 when this species was first established in Tasmania (Walker, 1988). Alternatively, as there has been no subsequent transfer of eggs or salmonid fish from New Zealand into Tasmania, transfer may have occurred through migratory fish or seabirds, which have been shown to be possible vectors (Willumsen, 1989). The serotype O2 isolates identified for the first time in Australia in this study are substantially different from O2 serotypes previously characterized in the USA, separated by more than 4000 SNPs when complete genome sequences are compared, which supports the proposition of separate evolution of multiple serotypes within Tasmania. The O2 variant is characterized by the complete absence of the O-antigen cluster. This deletion appears to have occurred independently on three continents on three different occasions, evident as quite distinct genetic lineages of the isolates with MRCA dates estimated at 16 923 YBP (HPD 11 456–25 237), 15 717 YBP (100 441–23 827) and 13 634 YBP (9407–20 237). The LPS O-antigen has been shown to be both necessary and type-specific for adaptive immune protection against *Y. ruckeri* infection in salmonids (Welch & LaPatra, 2016). Consequently, both naturally acquired and vaccine-induced adaptive immunity amongst host fish populations are likely to confer strong selective advantage on O-antigen-deficient types of *Y. ruckeri*, although estimated divergence dates for the independent losses clearly support naturally acquired immunity as a driver. To date, there is no evidence of transfer of the O1a (Hagerman) strain into Tasmania or New Zealand, suggesting that continuing inward biosecurity surrounding importation of genetic material or raw salmonid products has been effective.

The absence of the O1a serotype and the presence of a genetically distinct O2 serotype are noteworthy given the origins of salmonid stock in Australasia. The first successful introduction of salmonids to Australia was to Tasmania with the hatching of eyed-ova of brown trout (*Salmo trutta*) and Atlantic salmon in 1864 (Walker, 1988). The ova were collected from wild stock in the UK and packed on beds of moss kept cool and moist with water from melting ice. As non-migratory species, brown trout became well established in Tasmania but Atlantic salmon, being migratory, did not (Gilmour, 1996). Atlantic salmon for Tasmania’s current aquaculture industry were derived from landlocked stock held in New South Wales. These fish were obtained from the Phillip River in Nova Scotia, Canada, between 1963 and 1965 (Jungalwalla, 1991) and, following extensive testing, eggs were transferred to Tasmania between 1984 and 1986 as the foundation stock for fish farming. Since 1986 there have been no importations of genetic stock to Tasmania. Establishment of rainbow trout in Tasmania occurred in 1898 with eggs obtained from New Zealand (Walker, 1988), which obtained its progeny in 1883 from steelhead trout from Sonoma Creek in northern California (Scott *et al.*, 1978). Chinook salmon are the only commercially farmed species of salmonid in New Zealand. These fish were introduced from the Baird River hatchery on the McCloud River, a tributary of the Sacramento River, in California in the late 1800s and early 1900s (McDowall, 1994), but there have been no further imports of this species into New Zealand since 1907. Further species of salmonids were also introduced during this time originating from California (rainbow trout), Tasmania (brown trout), Canada (sockeye and Atlantic Salmon), England, Scotland and Germany (Atlantic salmon) with the last imports in 1910 (Haworth, 2010).

Despite the European and North American origins of Australasian salmonid stocks, it is of note that the lineages of *Y. ruckeri* extant in Australia and New Zealand are quite distinct from those of the northern hemisphere. This suggests that *Y. ruckeri* in Australia and New Zealand are pre-existing ancient strains that have emerged following the introduction of susceptible hosts. Salmonids are not native to Australasia, although salmoniformes of the Galaxiidae, Lepidogalaxiidae and Retropinnidae do occur. Whether fish of this type are natural hosts of *Y. ruckeri* has not be established but it is known that *Y. ruckeri* does occur in non-salmonid fish (reviewed by Barnes, 2011).

At around 3.8 Mbp, the *Y. ruckeri* genome is substantially smaller (10–15 %) than other members of the genus *Yersinia* sequenced to date (ranging from 4.27 to 4.86 Mbp) (Chen *et al.*, 2010; Reuter *et al.*, 2014). This is consistent with smaller genomes found in aquatic bacterial species, perhaps a result of improved fitness in an oligotrophic environment outside the host. Indeed, genomes of aquatic isolates of *Streptococcus agalactiae* are around 10 % smaller than those of their terrestrial conspecifics (Rosinski-Chupin *et al.*, 2013). In both *Y. ruckeri* and *S. agalactiae*, reduced metabolic capability compared to most closely related terrestrial isolates explains the majority of the genome depletion (Reuter *et al.*, 2014; Rosinski-Chupin *et al.*, 2013).

Assembly of a pangenome and subsequent analysis of gene presence and absence using the GVIEW server and Roary, respectively, revealed that the majority of differences amongst the isolates analysed was explained by presence or absence of putative prophage and plasmids. The abundance of prophage may result from the lack of CRISPR-*cas* regions in any of the *Y. ruckeri* genomes sequenced. This is in contrast to other pathogenic members of the genus *Yersinia*, *Y. pestis* and *Y. pseudotuberculosis,* that have well-characterized CRISPR clades (Barros *et al.*, 2014; Koskela *et al.*, 2015), but similar to *Y. enterocolitica* subsp. *palearctica* that also completely lacks CRISPR regions (Leskinen *et al.*, 2016). The fitness cost/benefit of CRISPR-based immunity versus phage susceptibility depends upon both the availability of other mechanisms of phage immunity and the dependence of the host bacterium on mobile genetic elements for survival (Marraffini, 2013). In *Y. ruckeri*, the high number of prophage and plasmid-associated genes that fulfil metabolic roles in a genome that is otherwise substantially reduced in metabolic capability compared to *Y. pestis* and *Y. pseudotuberculosis* (Reuter *et al.*, 2014) may explain why the absence of a CRISPR-*cas* system is advantageous; genes carried by mobile genetic elements would not be able to coexist with matching fragments contained in a CRISPR-*cas* spacer region (Marraffini, 2013). *Y. ruckeri* may use other mechanisms to prevent phage-mediated lysis, such as employment of restriction modification systems. Indeed, multiple restriction endonucleases were detected amongst the *Y. ruckeri* genomes.

Recombinant *Y. ruckeri* flagellin initiates a rapid inflammatory response through induction of expression of specific IL12 isoforms via TLR5 in rainbow trout (Wangkahart *et al.*, 2016). Consequently, expression of flagella can decrease fitness during colonization and dissemination within the host. This selective signal has led to evolution of non-motile phenotypes of several pathogens (Haiko & Westerlund-Wikström, 2013; Minnich & Rohde, 2007). For example, an aflagellate phenotype is highly prevalent in pathogenic isolates of *Salmonella enterica* serovar Dublin and is associated with reduced inflammatory response in the gut and systemic dissemination through human hosts (Yim *et al.*, 2014). In *Y. ruckeri*, the non-motile biotype 2 has arisen independently in the USA and UK and on multiple occasions in continental Europe (Welch *et al.*, 2011; Wheeler *et al.*, 2009) through mutations in different genes in the 60+ gene flagellar biosynthesis cluster. In Tasmania, the biotype 2 phenotype has arisen independently through a frameshift in the 454 aa FliI flagellum-specific ATP-synthase in isolates QMA0427, 431, 432 and 433. Read mapping identified a deletion of G at nucleotide position 242 resulting in an early stop codon at 421–433 and two ORFs of 140 aa at the N-terminal end and 313 aa at the C-terminal end containing the walkerA and walkerB motifs and the ATP binding domains. Both of the ORFs are expressed in culture, confirmed by RT-PCR, but the frameshift is likely to render the ATPase non-functional, as the key active domains become nonsense. Because the ATPase also drives the export of the flagellin through the type III secretion system basal complex, this mutation alone is sufficient to explain the lack of flagella in the biotype 2 strains shown by TEM.

Biofilm formation is critical in *Y. ruckeri* epidemiology and persistence in the fish farm environment, yet biofilm formation by *Y. ruckeri* has been correlated with high flagella motility (Coquet *et al.*, 2002). Conversely, motility of the O1b lineage in Tasmania has decreased since 2002, with a lower motility lineage, which also includes the non-motile biotype 2 isolates, currently persisting amongst isolates from salmon epizootics. All of the O1b genomes contain the *mrf* pilin export gene cluster similar to the *mrk* cluster in *Klebsiella pneumoniae* that is critical for biofilm formation (Schroll *et al.*, 2010; Wilksch *et al.*, 2011). In *K. pneumoniae*, the *mrf* pilus cluster was absent from the serotype O1a strains (ATCC 29473, CSF007-82 and RS41), although both ATCC 29473 and CSF007-82 harbour a plasmid with complete apparatus for synthesis and export of Type IV pili, which have been shown to be important for biofilm formation in many enterobacterial species (Craig *et al.*, 2004). It may be that the lack of the *mrf* pilus cluster in the O1a serotype accounts, at least in part, for the antigenic difference between the O1a and O1b serotypes that share the same LPS O-antigen cluster.

Tasmania and New Zealand are geographically isolated and have no native salmonid population, but brown trout, rainbow trout and Atlantic salmon eggs were imported for sport and food fishery from around 150 years ago, and in the last 30 years a salmonid farming industry has been established. The serotype O1b Australasian isolates of *Y. ruckeri* are genetically distinct from any other global isolates and belong to the O1b serotype, which has not been reported elsewhere apart from a relatively recent isolation in Chile. For the first time, we have also demonstrated the presence of an O2 serotype in Tasmania, but this too has a completely distinct genetic origin to the O2 serotype initially identified in the USA, supporting our contention that *Y. ruckeri* probably pre-existed in the region well before European colonization and has evolved independently.

## Supplementary Data

2204 Supplementary File 1
