# Supplementary material for: Whole genome analysis of Yersinia ruckeri isolated over 27 years in Australia and New Zealand reveals geographical endemism over multiple lineages and recent evolution under host selection
Source: Microb Genom. 2016 Nov 30;2(11):e000095. doi: 10.1099/mgen.0.000095 (PMC5320707; doi:10.1099/mgen.0.000095)

Supplementary Table 1. Primers used in this study

Primer	Sequence 5'-3'	Tm °C	Amplicon size (kb)	Comments
10897F	cttcggcagtgacataaccg	65.5	10.7	QMA0440 gap closing PCR using LongAmp *Taq* (New England Biolabs). After initial denaturation at 94°C for 30 sec, cycling for 30 cycles of 94°C 30 sec; 60°C 30 sec; 65°C 10 min, and final extension of 10 min.
21587R	gtctccacgcatcaccattc	65.6		
8421F	agcatcaggaagcagaaggt	62.9	11.2	
19453R	caggtgcaatacggtgatgg	66.3		
21568F	gaatggtgatgcgtggagac	65.6	11.1	
32676R	ctagtgtcgaatgtgccacg	64.4		
-3515F	gctgtcgtcttctgcatcag	63.9	7.4	
3928R	gcatgctggtgttatgcgta	64.6		
-5163F	ccaacggcctttatacaccg	66.1	7.8	
2743R	gttcggcggggtacataaac	65.1		
-4697F	ccaatccgctcaccaatctg	67.8	6.9	
2342R	atgtggatggcgttaatggc	66.7		
-5484F	cctgactcatccgggatctc	66.0	7.9	
2457R	aaactgaatggcaaggcgtt	65.6		
-4354F	gcaacgccctaccaaagaaa	66.0	7.2	
2994R	aaatccgttgcgactaaccg	65.9		
FliH_F	agtttgatggcgcagaagat	63.7	0.2	Flagella operon RT-PCR using MyTaq (Bioline). Initial denaturation 95°C then 30 cycles of 95°C 30 sec; 58°C 30 sec; 72°C 30 sec; final extension 72°C 1 min. FliC primers were multiplexed.
FliH_R	gcttccagcataccttgctc	63.8		
FliI_indF	aagtgcaggaagtggaaagc	63.3	0.2	
FliI_indR	ataaccggtttctggtgctg	63.8		
FliI_longF	cgtggtcgtgaagtcaaaga	64.0	0.2	
FliI_longR	gcgggtcagagaatccataa	63.9		
FliI_shortF	gcgcctatttctgatgcact	64.2	0.2	
FliI_shortR	ataaccggtttctggtgctg	63.8		
FliJ_F	ttgttactctgcgcgatttg	63.8	0.2	
FliJ_R	tgctgtttctcttgccaatg	64.0		
FliC1541F	ctaatgatgcggctgtggtc	65.5	0.267	
FliC1808R	ttgccattaaccactgcacc	65.7		
FliC4111F	cgttccactagtgcgtcatg	64.4	0.657	
FliC4768R	gtcgttattcagacgggcac	64.4		

Supplementary Table 2: Draft assembly statistics (Quast v4.2) for SPAdes 3.7.1 assembly of Illumina paired end reads derived from genomic DNA Australian and New Zealand *Y. ruckeri* isolates

Assembly	# contigs (>= 0 bp)	# contigs (>= 1000 bp)	# contigs (>= 5000 bp)	# contigs (>= 10000 bp)	# contigs (>= 25000 bp)	# contigs (>= 50000 bp)	Total length (>= 0 bp)	Total length (>= 1000 bp)	Total length (>= 5000 bp)	Total length (>= 10000 bp)	Total length (>= 25000 bp)	Total length (>= 50000 bp)	# contigs	Largest contig	Total length	GC (%)	N50	N75	L50	L75	# N's per 100 kbp
QMA0397	91	49	30	27	24	20	3736859	3719549	3673392	3646800	3597206	3446376	63	523172	3728769	47.63	195994	114296	7	13	0
QMA0398	88	52	32	28	25	21	3737587	3722739	3678852	3646641	3597047	3446283	64	523172	3730320	47.62	170601	114296	7	13	0
QMA0399	92	52	33	29	26	22	3735979	3718856	3676560	3644349	3596916	3446152	66	411926	3728187	47.62	170601	111091	7	14	0
QMA0400	89	52	34	29	26	22	3736436	3720407	3678847	3640334	3590740	3439976	65	523172	3729072	47.63	170601	108701	7	14	0
QMA0401	73	47	37	33	28	23	3762398	3751085	3731151	3705082	3607871	3428883	56	359959	3757676	47.58	170486	113877	8	15	0
QMA0402	97	55	34	30	27	23	3736833	3720516	3672521	3640310	3592877	3446190	68	411926	3728723	47.62	167633	108498	8	15	0
QMA0403	93	52	34	30	27	23	3737321	3720191	3675736	3643525	3593931	3447244	66	412136	3729427	47.63	167633	108463	8	15	0
QMA0404	89	51	33	29	26	22	3736724	3720864	3679091	3646880	3597286	3446522	64	412230	3729217	47.63	170601	111301	7	14	0
QMA0405	86	52	33	29	26	22	3738811	3723877	3680981	3648770	3598972	3447880	63	412165	3731722	47.62	170601	111327	7	14	0
QMA0406	91	53	32	28	26	22	3647605	3631826	3587284	3555073	3529743	3378785	65	412100	3639754	47.64	167633	111793	8	14	0
QMA0407	88	51	33	28	26	22	3740373	3724231	3686465	3648644	3623314	3472356	63	523183	3732840	47.63	167633	108474	7	14	0
QMA0408	128	73	38	31	27	23	3809302	3787654	3707897	3657111	3597217	3457386	91	523127	3799376	47.63	146794	94472	8	15	0
QMA0409	50	48	33	28	26	22	3720670	3719881	3682065	3641782	3616452	3465494	48	412212	3719881	47.63	170601	111301	7	14	0
QMA0410	85	54	34	28	24	21	3758885	3745150	3701930	3656144	3600372	3479407	65	523174	3752948	47.64	170601	108594	7	14	0
QMA0411	82	53	32	27	25	21	3739307	3726741	3681222	3640939	3615609	3464651	63	523154	3733481	47.63	170601	108677	7	14	0
QMA0412	79	59	36	30	26	23	3756563	3746078	3699775	3654083	3598404	3477433	67	412185	3752095	47.64	167633	108594	8	15	0
QMA0413	79	52	35	29	27	23	3740714	3727856	3690269	3640223	3612465	3460997	66	309719	3737462	47.63	168506	108416	8	15	0
QMA0414	86	52	33	27	25	22	3739716	3725509	3685610	3639025	3613695	3504610	63	523109	3732920	47.62	203109	108594	7	14	0
QMA0415	106	64	42	36	32	26	3763070	3748101	3699311	3650635	3590790	3372699	77	309719	3757104	47.63	138185	89270	9	18	0
QMA0416	99	60	41	36	32	24	3772562	3762944	3725993	3688944	3606092	3333869	66	312848	3767117	47.59	147047	87347	9	17	0
QMA0417	106	57	38	31	27	23	3746724	3732189	3690476	3631735	3562737	3421629	70	309719	3740912	47.62	186952	108535	8	15	0
QMA0418	134	81	46	37	31	25	3811679	3791689	3710373	3638678	3536984	3339188	102	309719	3804971	47.63	143343	81990	9	18	0
QMA0419	90	57	38	33	29	24	3743328	3730268	3691952	3651669	3598982	3414964	71	309719	3739594	47.63	168506	99282	9	16	0
QMA0420	102	62	43	38	31	24	3763214	3748279	3707577	3665550	3565715	3316285	77	309719	3758355	47.64	146794	94433	9	17	0
QMA0421	86	57	37	32	27	23	3761436	3748800	3705392	3663673	3581834	3437824	70	309719	3757491	47.63	186542	111091	8	15	0
QMA0422	119	66	43	34	32	25	3744290	3728139	3675108	3601640	3573493	3300047	80	309719	3737694	47.62	134906	86364	10	18	0
QMA0423	97	57	37	32	28	25	3761900	3746344	3702284	3660436	3592446	3480860	71	309719	3756030	47.64	168506	99287	8	16	0
QMA0424	87	65	47	39	32	24	3810988	3800686	3760364	3697202	3569831	3283311	77	315280	3808805	47.59	144904	81901	10	18	0
QMA0425	130	77	45	38	30	24	3812161	3791285	3718870	3661169	3524604	3314524	100	309725	3806640	47.63	137958	94363	9	17	0
QMA0426	83	56	33	29	26	23	3762041	3749086	3699839	3663472	3619748	3508293	68	309719	3757695	47.64	168506	111819	8	15	0
QMA0427	99	59	40	35	29	25	3759139	3744144	3701321	3661092	3568224	3424103	73	309719	3753379	47.64	143513	94440	9	17	0
QMA0428	84	55	35	31	27	24	3762322	3750139	3706184	3673974	3616379	3505341	67	309719	3758096	47.63	146794	108416	9	16	0
QMA0429	90	57	36	32	28	25	3762511	3748765	3701635	3664829	3596862	3485406	70	323342	3758224	47.64	143997	108247	9	16	0
QMA0430	92	59	37	33	28	24	3762002	3748910	3699837	3663590	3589378	3453890	72	309718	3757721	47.63	168506	99288	8	16	0
QMA0431	94	53	36	32	26	23	3758913	3742510	3702942	3666695	3570293	3458823	70	309719	3753812	47.64	186542	111678	8	15	0
QMA0432	93	57	37	33	28	24	3758422	3745057	3701459	3665227	3593090	3449079	70	309719	3753686	47.64	168506	108416	9	16	0
QMA0433	88	56	38	34	30	25	3759996	3746331	3701461	3664640	3606374	3423762	69	309719	3755476	47.64	146794	89270	9	17	0
QMA0434	135	66	48	40	31	24	3765476	3744613	3702324	3639818	3509070	3241438	85	311029	3757615	47.64	129165	69538	9	18	0
QMA0435	125	68	40	36	31	25	3819581	3795110	3732882	3706050	3624700	3411950	97	309763	3813436	47.64	134247	93630	10	18	0
QMA0436	76	46	30	27	25	18	3741050	3731187	3703079	3679645	3639981	3399232	54	525714	3736780	47.56	252260	124942	6	12	0
QMA0437	86	46	31	28	26	19	3741014	3730549	3702647	3679347	3638606	3386745	55	525203	3736300	47.56	186581	127077	7	13	0
QMA0438	87	48	33	30	28	20	3741241	3727488	3702079	3678779	3639117	3354824	62	525372	3736750	47.56	185521	119916	7	14	0
QMA0439	93	62	38	33	29	25	3783539	3771599	3714863	3673633	3614037	3476338	73	271349	3779262	47.63	140378	111586	10	17	0
AHL1	77	50	37	36	29	26	3861398	3851156	3823689	3816306	3694479	3593351	60	359170	3857563	47.6	144122	93624	9	16	0
AHL2	106	56	39	30	28	22	3690018	3673541	3637974	3565877	3532560	3297157	69	389804	3681627	47.56	155481	90756	8	15	0
AHL3	79	49	39	38	31	24	3897373	3886187	3863099	3855716	3733785	3501274	60	316303	3893794	47.61	186473	86869	9	16	0
AHL4	70	42	32	27	25	20	3711371	3703531	3678493	3638434	3604800	3428030	49	521428	3707941	47.58	255492	94399	6	12	0
[illegible]	[illegible]	[illegible]	[illegible]	[illegible]	[illegible]	[illegible]	[illegible]	[illegible]	[illegible]	[illegible]	[illegible]	[illegible]	[illegible]	[illegible]	[illegible]	[illegible]	[illegible]	[illegible]	[illegible]	[illegible]	[illegible]

Supplementary Table 3. Antisera, reference isolates used in serotyping and macroscopic agglutination assay results

Yersinia ruckeri Control Isolates

SFC 37 | ATCC 29473 | Serotype O1a |Originally isolated by Ewing
Y. ruckeri | Rainbow trout (*Oncorhynchus mykiss*) with Red Mouth Disease, USA

SFC 47 | 88:8181-5A | Serotype O1b |Originally isolated by N. Gudkovs
Y. ruckeri | Atlantic salmon (*Salmo salar*) bacterial septicaemia, Benalla, Victoria, Australia; 1988
SFC 124 | RD6 | Serotype O1|Clone 2|Biotype 2|OMP-type 1|Originally isolated by R.L. Davies
Y. ruckeri | Rainbow trout | AAHL <--- R.L. Davies
SFC 127 | RD34, BA24 | Serotype O2|Biotype 1|OMP-type 1|Originally isolated by B. Austin
Y. ruckeri | Brown trout (*Salmo trutta*)| AAHL <--- R.L. Davies

Antisera (rabbit)

**Serum**	**Specificity**
	**Serotype O1** | SFC 124 |isolate RD6 from Davies
**R452-1**	*Serum against whole cell antigen from serotype O1 (Davies)*
**R452-6**	Serotype O1 specific - *Serum against boiled cell antigen from serotype O1 (Davies RD6)*
	**Serotype O2** | SFC 127 | isolate RD34 from Davies
**R452-2**	*Serum against whole cell antigen (Davies RD34 serotype O2)*
**R452-12**	Serotype O2 specific - *Serum against phenol extracted LPS antigen, proteinase-K digested (Davies RD34)*

Macroscopic agglutination (units are arbitrary)

CONTROL

Antiserum

**Isolate**	R452-1 (O1)	R452-2 (O2)	R452-6 (O1)	R452-12 (O2)
SFC 37 (O1a)	++	-	++	-
SFC 47 (O1b)	+	-	++	-
SFC 124 (O1)	++	-	+++	-
SFC 127 (O2)	-	+++	-	+++

TEST

**Isolate**	R452-1 (O1)	R452-2 (O2)	R452-6 (O1)	R452-12 (O2)
QMA0436	-	+++	-	+++
QMA0437	-	+++	-	+++
TCFB 3030	-	+++	-	+++
TCFB 3033	-	+++	-	+++

TCFB: Tasmanian Collection of Fish Bacteria (DPIPWE)

Supplementary Table 4. Gene annotations in the *Y. ruckeri* O-antigen biosynthesis cluster with positions in the reference genome.

Gene	Size (aa)	Size (nt)	Function	Position in QMA0440 genome	
tetR	192	576	transcriptional regulator tetR family	3492534	3493109
tRNA-phe		75	transfer RNA Phe(gaa)	3493251	3493326
wbuC	134	402	metalloprotein	3493560	3493961
wbuB	402	1206	putative glycosyl transferase	3493952	3495157
qnlB1	377	1131	UDP-N-acetylglucosamine 2-epimerase	3495157	3496287
rmlC	368	1104	NAD-dependent epimerase/dehydratase family protein	3496287	3497390
ddhB	345	1035	CDP-glucose 4,6-dehydratase/5-epimerase	3497392	3498426
wbuZ	265	795	amidation-associated protein	3498439	3499233
wbuY	209	627	glutamine amidotransferase	3499226	3499852
wbuX	378	1134	N-acetyl sugar amidotransferase	3499849	3500982
wbuW	406	1218	putative glycosyl transferase	3500969	3502186
wzz	425	1275	chain length determinant	3502209	3503483
wzx	413	1239	O-antigen flippase	3503568	3504806
idnO	255	765	gluconate 5-dehydrogenase	3504833	3505597
neuA	229	687	CMP-N,N'-diacetyllegionaminic acid synthase	3505610	3506296
afr	321	963	1,5-anhydro-D-fructose reductase	3506298	3507260
ddhA	352	1056	glucose-1-phosphate cytidylyltransferase	3507263	3508318
wbuL	212	636	Putative acetyltransferase	3508339	3508974
neuB	358	1074	CMP-N-acetyl-neuraminic acid synthase	3508967	3510040
qnlB2	389	1167	UDP-N-acetylglucosamine 2-epimerase	3510047	3511213
degT	384	1152	UDP-4-amino-4-deoxy-L-arabinose--oxoglutarate aminotransferase	3511210	3512361
gne	397	1191	UDP-N-acetyl-alpha-D-glucosamine C6 dehydratase	3512377	3513567
YedF	80	240	SirA-like protein/sulphurtransferase	3514623	3514862
YedE	358	1074	putative inner membrane protein sulphur transport	3514999	3516072
gltP2	439	1317	glutamate/aspartate:proton symporter	3516353	3517669
acs	653	1959	acetyl-CoA synthetase	3518596	3520554

Supplementary Figure 1 Maximum likelihood phylogeny and regions of recombination identified amongst Y. ruckeri isolates with Gubbins

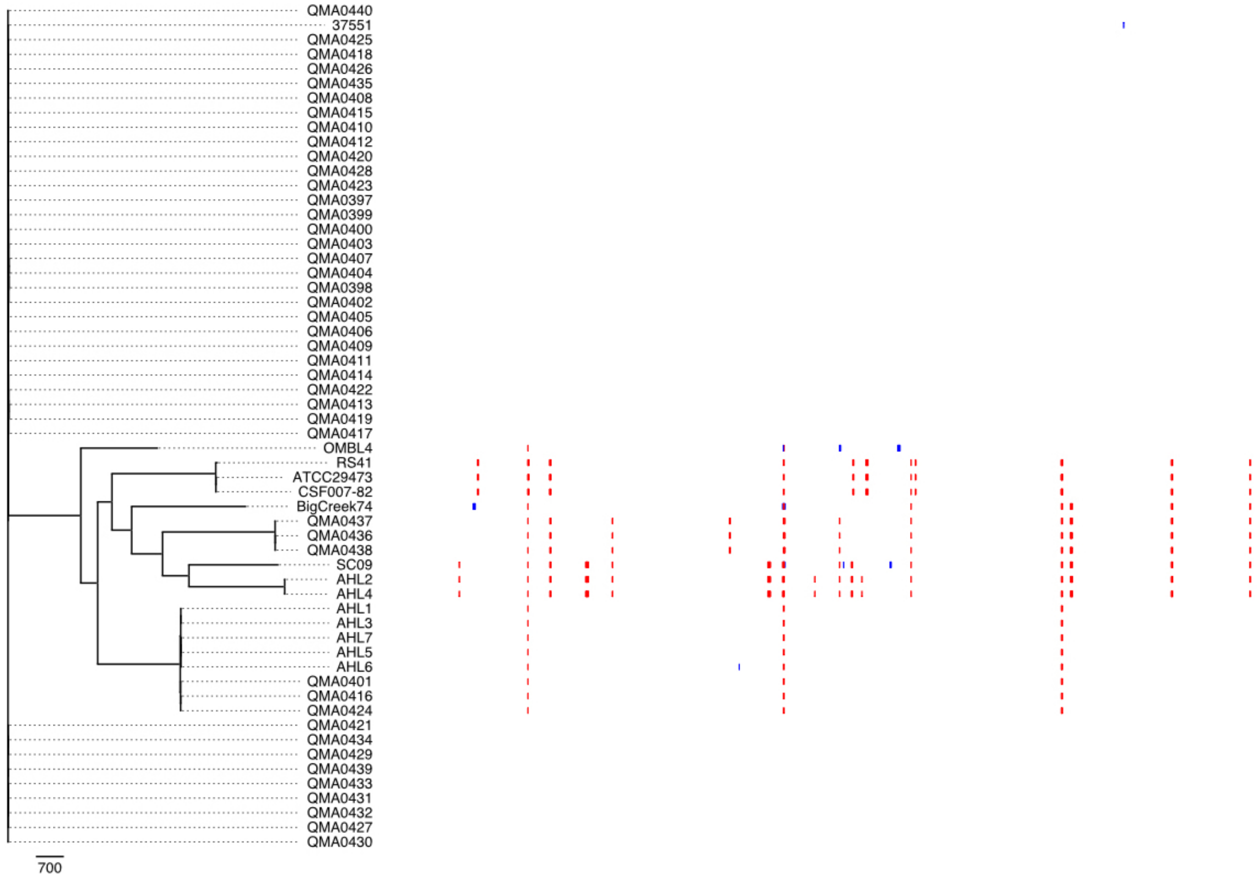

Supplementary Figure 2. Root to tip regression of substitutions against time (date of isolation) using TempEst. Regression was performed on trees constructed in RAxML by maximum likelihood analysis of alignments of core-genome SNPs after recombination filtering using Felsentein's correction for ascertainment bias (See text). Where date of isolation was unknown, data were excluded from the analysis. Where SNP profiles for isolates were identical, one representative was retained in the analysis. A) Set of Australasian and exotic isolates isolates (n=42): Substitution rate = 1.10×10^{-6}, correlation (Pearson) 0.5224, $R^2 = 0.2729$, residual mean = 4.56×10^{-10}. B) Atlantic salmon serotype O1b isolates (n=38) from Tasmania: Substitution rate = 4.87×10^{-7}, correlation = 0.827, $R^2 = 0.6846$, residual mean = 6.25×10^{-12}.

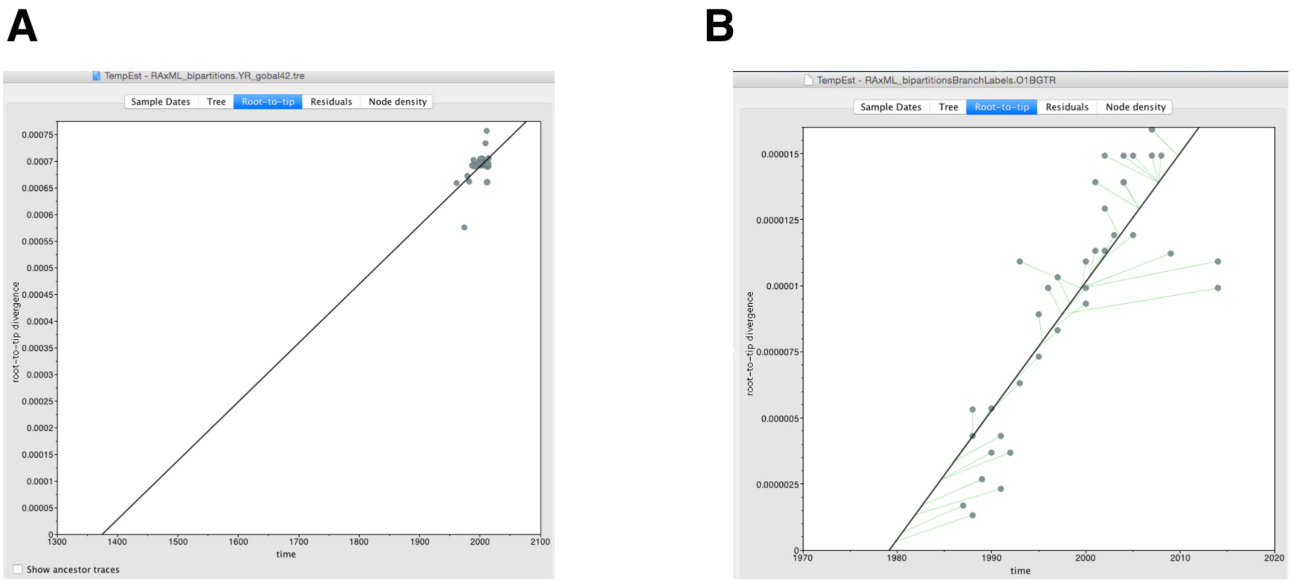

Supplement: Supplementary File 1 [file mgen-02-95-s001.pdf]
